# The presence and potential impact of psychological safety in the healthcare setting: an evidence synthesis

**DOI:** 10.1186/s12913-021-06740-6

**Published:** 2021-08-05

**Authors:** K. E. Grailey, E. Murray, T. Reader, S. J. Brett

**Affiliations:** 1 Department of Surgery and Cancer, Imperial College London, London, UK; 2 Said Business School, University of Oxford, Oxford, UK; 3 Department of Psychological and Behavioural Science, London School of Economics and Political Science, London, UK

**Keywords:** Psychological safety, Qualitative research, Healthcare workers

## Abstract

**Introduction:**

Psychological safety is the shared belief that the team is safe for interpersonal risk taking. Its presence improves innovation and error prevention. This evidence synthesis had 3 objectives: explore the current literature regarding psychological safety, identify methods used in its assessment and investigate for evidence of consequences of a psychologically safe environment.

**Methods:**

We searched multiple trial registries through December 2018. All studies addressing psychological safety within healthcare workers were included and reviewed for methodological limitations. A thematic analysis approach explored the presence of psychological safety. Content analysis was utilised to evaluate potential consequences.

**Results:**

We included 62 papers from 19 countries. The thematic analysis demonstrated high and low levels of psychological safety both at the individual level in study participants and across the studies themselves. There was heterogeneity in responses across all studies, limiting generalisable conclusions about the overall presence of psychological safety.

A wide range of methods were used. Twenty-five used qualitative methodology, predominantly semi-structured interviews. Thirty quantitative or mixed method studies used surveys.

Ten studies inferred that low psychological safety negatively impacted patient safety. Nine demonstrated a significant relationship between psychological safety and team outcomes.

The thematic analysis allowed the development of concepts beyond the content of the original studies. This analytical process provided a wealth of information regarding facilitators and barriers to psychological safety and the development of a model demonstrating the influence of situational context.

**Discussion:**

This evidence synthesis highlights that whilst there is a positive and demonstrable presence of psychological safety within healthcare workers worldwide, there is room for improvement. The variability in methods used demonstrates scope to harmonise this. We draw attention to potential consequences of both high and low psychological safety.

We provide novel information about the influence of situational context on an individual’s psychological safety and offer more detail about the facilitators and barriers to psychological safety than seen in previous reviews. There is a risk of participation bias - centres involved in safety research may be more aligned to these ideals. The data in this synthesis are useful for institutions looking to improve psychological safety by providing a framework from which modifiable factors can be identified.

**Supplementary Information:**

The online version contains supplementary material available at 10.1186/s12913-021-06740-6.

## Introduction

Healthcare workers are required to operate in challenging and fast paced environments, where accurate decision making, error minimisation and innovation are essential in providing excellent patient care [1, 2]. Psychological safety was originally defined in 1990 as an individual’s “sense of being able to show and employ oneself without fear of negative consequences to self-image, status or career” [3]. Psychological safety has been characterised further in the context of work teams as “a shared belief that the team is safe for interpersonal risk taking” [4].

An environment that is psychologically safe allows individuals to be their “true selves”. This can take the form of enhancing employee voice, commitment to the organisation and investment in patient care [5]. An individual that feels enabled to raise concerns, near misses and difficult issues can also help minimise the incidence of medical error [6, 7].

The importance of psychological safety is not limited to the healthcare setting. Google explored this concept within “Project Aristotle” [8] – a 2-year project investigating the factors that made teams operate most effectively (exploring group dynamics, individual skill sets, personality traits and emotional intelligence). From this they developed a list of key dynamics making teams successful - with psychological safety at the top [8]. In industry, high levels of psychological safety can be associated with promoting moderate risk taking and creative breakthroughs – for example, during product development new ideas can be proposed without fear of criticism [9–11]. It is essential in maintaining safety (construction workers highlighting scenarios that may result in injury) and encouraging improvement [12]. Within the healthcare setting it promotes the ability to speak up - minimising poor practice and medical error [13]. There are additional benefits to a psychologically safe environment within the healthcare setting. These include an improvement in wellbeing, reduction in work related stress, an understanding of the importance of learning from failures and an increased engagement in quality improvement [14]. Psychological safety is an important antecedent to quality improvement as it allows the open sharing of operational failures [15] and facilitates productive discussion [16]. This enables the development of solutions that prevent repeated occurrences of errors through the creation of organisational memory, rather than individuals creating a work-around without communicating the issue to the rest of the team (leading to a risk that the error may be repeated) [17, 18]. A recent systematic review [12] of the safety voice literature highlights that in healthcare workers, “employees report a hesitancy for raising safety concerns”. A 2014 review [19] makes three key conclusions about psychological safety (its “role in enabling performance”, its “relevance for understanding organisational learning” and its presence making individuals “more likely to speak up at work”). This review also highlights areas for future research – including exploring the factors that promote or reduce psychological safety.

Traditionally, healthcare teams have operated under a strict hierarchy [20]. The presence of a professional hierarchy is well established within the healthcare setting and has been recognised as a barrier to psychological safety - with those in higher positions having increased freedoms to speak and be themselves [21]. This can prevent individuals in lower positions from speaking across professional boundaries and may subsequently reduce the opportunity for collaborative learning and error reduction [22]. Whilst much work has been done to flatten this (through dedicated non-technical skills training [23] and improvement of communication skills [24]) it is still a contributing factor to medical error [25]. The importance of psychological safety within the healthcare setting should not be underestimated. Psychological safety is important because it allows those in junior positions within the professional hierarchy (often the individuals most acutely aware of potential safety issues) to speak up. A lack of psychological safety as a result of such a hierarchy can inhibit the communication of problems and creative solutions from those in junior positions who witness them to those higher up within the organisation. This limits the potential for organisational learning [17]. The presence of psychological safety fosters a culture where healthcare workers will raise safety concerns as they arise because they aren’t concerned about the potential consequences. In such a culture an individual will feel confident that the organisation will listen to and act upon such concerns, irrespective of who within the “hierarchy” raises it.

Medical error rates remain high both within the UK and worldwide [26, 27]. In addition, healthcare staff report ongoing dissatisfaction with their working environment– a recurring theme within the annual NHS staff survey [28]. The delivery of exemplary healthcare requires multiple skill sets – as a result healthcare teams comprise individuals with specific roles and skills. Consequently, a good understanding of each other’s strengths and weaknesses is essential. It is known that a high proportion of medical errors have poor communication as a causative element (a 2015 report on malpractice claims in the US [29] implicated communication failure in 30% of all malpractice claims and 37% of high severity injury cases). Teams in the healthcare setting are interprofessional, relying upon a shared team identity and a collective understanding of each other’s roles and responsibilities [30]. The interprofessional nature of these teams can comprise of multiple differing interests and opinions that may create challenges in the absence of good communication [14]. Effective communication within the interprofessional team is facilitated by team psychological safety, allowing collaborative decision making [31]. Since high psychological safety is a promotor of good communication within teams [32] (allowing those with differing aims and working practices to communicate and work together successfully [19]), the benefit of this review lies in its potential to further understand how psychological safety has been explored within the clinical literature, looking at the importance of psychological safety by evaluating its role in shaping behaviour across multiple studies, the mechanisms through which psychological safety shapes behaviour and identifying future research needs. Namely – what is “normal”, how has it been measured, and whether psychological safety really is important. To address these aims, this study employs thematic analysis, content analysis and evidence synthesis (encompassing all research methodologies – quantitative, mixed methods and qualitative data) - techniques used in similar qualitative syntheses on quality in healthcare [33].

High levels of psychological safety have clear benefits for patient safety by improving the delivery of clinical care. In addition, it also improves the health of the workforce by promoting job satisfaction & well-being [34, 35]. Previous studies into psychological safety tend to focus upon outcomes in terms of patient safety or organisational productivity, without looking at the experiences of the healthcare workers themselves. This study aimed to keep these staff experiences at the centre of the analysis.

There are widely used tools for the assessment of psychological safety [4, 36], but it is unclear which ones are preferred and how frequently they are used in studies on healthcare workers.

There were three key objectives within this evidence synthesis:

- Objective 1. Synthesise existing literature investigating psychological safety in healthcare workers and use qualitative research methods to explore the presence of psychological safety in this workforce.
- Objective 2. Identify the methods used to assess psychological safety in healthcare workers.
- Objective 3. Review the literature for evidence of consequences of high or low psychological safety.

## Methods

The study protocol was developed using the EPOC (Cochrane Effective Practice and Organisation of Care Group) template [37] and registered on Prospero (https://www.crd.york.ac.uk/prospero/ Registration Number: CRD42019120104).

The study protocol was initially designed as an evidence synthesis with a focus upon qualitative research methods. The study design was intended (in line with qualitative research methodology) to evolve as an iterative process and following preliminary searches the inclusion criteria were expanded to include all studies exploring psychological safety in healthcare workers. This expansion occurred as it became clear that whilst a qualitative thematic analysis would address the first objective of this study, incorporating quantitative and mixed methods studies would allow a more comprehensive answer to the second two objectives of this synthesis to be developed.

A pre-planned comprehensive search strategy was subsequently developed with the aim of identifying all available studies addressing the topic of psychological safety in healthcare workers, either as a specified aim of the study or as a theme which emerged within the study analysis.

This evidence synthesis used PRISMA as its principle guideline [38]. As it was anticipated that a significant proportion of the included studies would utilise qualitative or mixed research methodology the Cochrane Qualitative and Implementation Methods Groups Guidance Series [38–43] were used in addition to structure the project. It is presented in accordance with ENTREQ (Enhancing transparency in the reporting of syntheses of qualitative research), a well cited tool which is included in the EQUATOR (Enhancing the QUality And Transparency Of health Research) network [44].

The SPIDER Tool [45] was used to define the plan for conducting this evidence synthesis and as the basis for the electronic search strategy & inclusion criteria.

### Sample

Healthcare workers (All members of the multidisciplinary team, all levels of seniority).

### Phenomenon of interest

Psychological Safety.

### Design

All primary studies that used qualitative study designs including ethnography, phenomenology, case studies, grounded theory studies and qualitative process evaluations. Studies that used qualitative methods for data collection (interviews, focus groups, observations and open-ended survey questions) and data analysis (e.g. thematic analysis) were included. Given the prevalence of surveys as a tool used to assess psychological safety, studies which were quantitative in their design were included. Studies were included irrespective of their publication status and language of publication.

### Evaluation

An exploration of the presence of psychological safety present in healthcare workers, the methods utilised to assess psychological safety, and the potential consequences of high or low psychological safety.

### Research type

Qualitative, Quantitative and Mixed methods.

#### Data sources

It is acknowledged both within the literature and published guidelines on the synthesis of qualitative studies that the indexing of published papers may be less robust than within quantitative databases. In order to capture as many qualitative studies that addressed the issue of psychological safety in healthcare workers as possible the search terms were kept deliberately broad. Complementary search strategies including citation searching, author searching, and reference list checking were also employed.

#### Electronic searches

The following electronic databases were searched to identify eligible studies for inclusion. Databases were searched from their date of origin through December 2018 (MEDLINE Ovid, Embase Ovid, PubMed, CINHAIL EBSCO Complete, Cochrane Library, Web of Science, Conference Proceedings Citations Index – Science, Global Health, Ovid, Google Scholar).

#### Search strategy

**S**: “Healthcare worker*” OR “Physician” OR “Nurs*” OR “Doctor” OR “Medic*”.

**PI**: “Psychological Safety OR Interpersonal Risk” OR “Team*” OR “Communication” OR “speak* up”.

**D**: “questionnaire” OR “Survey” OR “interview” OR “focus group” OR “case stud*” OR “obser*”.

**E**: “experience*” OR “opinion” OR “outcome*” OR “satisfaction”.

**R**: “qualitative” OR “mixed method” OR “quantitative”.

#### Selection of studies

Studies were initially reviewed by title (the deliberately wide search criteria allowed for rejection of many papers at this stage, as despite addressing teamwork or psychological safety they were clearly not related to the topic of interest – namely papers that did not address psychological safety within the healthcare setting.). Abstracts of potential papers for inclusion were reviewed for evidence that they addressed the topic of psychological safety, safety within healthcare, speaking up, or teamwork. This review and study selection were performed by one researcher in the team (KG).

Full text of papers deemed suitable for inclusion were retrieved and reviewed in depth. The methods with which psychological safety was assessed and the robustness and validity of this assessment was explored (using both the CASP Qualitative Checklist tool [46] and published guidance on the assessment of survey quality [47]). This was performed primarily by one researcher (KG), with discussion regarding suitability of papers for inclusion and imposed criteria for selection within the wider research team.

#### Data extraction

The following data were extracted from the included papers and assembled within an Excel table (Table 1) (Microsoft, Redmond, Washington, USA) to facilitate cross comparison and analysis.

**Table 1:** Data extraction categories

Category	Data Collected
Study Location	Country
Site	Hospital Department
	Clinical Environment
	Primary vs Secondary Care
Population of Healthcare Workers	Role
	Specialty
	Level of Seniority
Method of Sampling Participants	
Outcome Measures	Study Primary Outcome Measures
Research Methodology	Quantitative, Mixed, Qualitative
	Tools used to assess psychological safety
Measurement of Psychological Safety	Primary Outcome
	Secondary Outcome
	Theme that emerged within the results
Level of Psychological Safety Identified (if quantifiable)	If quantifiable: High / Low
	Supporting Evidence: Quotations & Data Extracts
Evidence of consequences of psychological safety	Evidence of effects on patients
	Evidence of effects on staff / the organisation

#### Assessment of the methodological limitations in included studies

Each qualitative study was reviewed for methodological limitations using the CASP Qualitative checklist tool [46]. The GRADE-CERQual (Confidence in the Evidence from Reviews of Qualitative Research) approach [40] was implemented to summarise our confidence in each finding. CERQual assesses confidence in the evidence based upon four key components: methodological limitations, coherence of the review finding, adequacy of the data contributing to a review finding and relevance of the included studies to the review question. It was anticipated a high proportion of quantitative or mixed methods studies would utilise surveys as a research strategy. The assessment of possible methodological limitations of these surveys was done in line with published guidance regarding survey quality [47–49].

#### Synthesis methodology

##### Objective 1

Exploration of psychological safety within each study’s participant group and synthesis of subsequent extracted data. The thematic analysis was primarily undertaken by one researcher (KG), with ongoing discussion with the wider research team at each stage.

This thematic analysis was completed in a three-step approach:

1. Familiarisation with the data and extraction of data related to psychological safety. Data included key concepts as derived by study authors and verbatim participant data from published manuscripts.
2. Coding of data related to a participant’s experiences of psychological safety and development of descriptive themes. To address the study objective, data that reflected a study participant’s psychological safety were coded into “evidence of high psychological safety” or “evidence of low psychological safety”. All extracted data were analysed to identify commonalities and report patterns in the data associated with the psychological safety within each study participant group (whether low, moderate or high). These patterns were developed into a hierarchical code structure.
3. Generation of analytical themes beyond the content of the original studies

##### Objective 2

Identification of methods used to assess psychological safety.

Data regarding the tools used to assess psychological safety were extracted from the methods section of each included study. These were grouped according to whether they were quantitative, qualitative or used mixed methodology. Within this, details regarding the exact method of data collection and analysis were extracted and coded.

##### Objective 3

Identification of any consequences of low or high psychological safety.

A content analysis approach was employed to identify any patterns in conclusions made by each study regarding the observed presence of psychological safety and possible outcomes. Data presented in results and discussion of each included study were reviewed and any data suggesting an association between psychological safety and a linked outcome were extracted and coded. The data were reported as a frequency of each possible consequence and the level of psychological safety it was related to (low or high). It was also recorded whether the conclusions regarding the consequence of psychological safety as identified in each study were the opinion / inference of the study authors or derived from statistical study data.

The datasets supporting the conclusions of this article are included within the article and its additional files.

#### Reflexivity

During the data synthesis the authors were aware of their own positions and reflected on how these may influence the study design, search strategy, inclusion decisions, data extraction, analysis, synthesis and interpretation of the findings.

For reflexivity, the positions of the authors are as follows: KEG is a PhD student with a clinical background in anaesthesia and critical care, TR is an academic in organisational and safety culture, EJM is a former NHS manager and is now an academic in organisational studies and SJB is a clinical academic and consultant in intensive care. All have prior experience with the conduct and analysis of qualitative studies in the healthcare environment.

At the outset of this review, all authors believed that individuals with high psychological safety would have higher job satisfaction and be less affected by stress within the clinical environment. The authors also believed that high levels of psychological safety would confer better teamworking and ultimately better outcomes for both the patient and the organisation. The team maintained a reflexive position throughout all stages of the review to minimise the risk that these presumptions would skew the analysis and subsequent interpretation of findings.

## Results

**Fig. 1:**
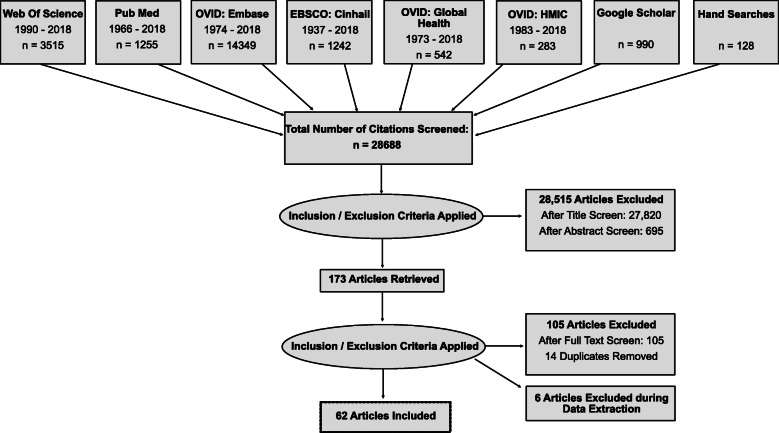
A PRISMA flow diagram demonstrating the search results and the process of screening and selection of studies for inclusion

As anticipated, by intentionally keeping the search strategy broad the number of papers retrieved by the initial database search was extensive with a total of 28,688 titles identified. This meant a huge number of studies (27,820) could be excluded at the title review stage as they were clearly unrelated to the research objectives. Abstracts of 868 papers addressing themes of teamwork, error reporting or psychological safety were reviewed and 173 were taken forward for full text review. Fourteen duplicates were removed, and 105 articles excluded following full text review. During data extraction a further 6 papers were excluded (these were either from the same research group and used data previously analysed in another study already included or did not provide sufficient data within the results section to use within a thematic analysis). Sixty-two papers were deemed eligible. The Prisma flow diagram for this process is illustrated in Fig. 1.

### Study characteristics

Study characteristics for each of the 62 included studies can be seen in Additional File 1. Included studies originated from 19 countries, encompassing a total of 32,677 participants. Sixty were hospital-based studies, with 2 incorporating both primary & secondary care sites. Forty-four papers assessed psychological safety as one of their primary outcomes, 3 listed it as a secondary objective and 15 papers discussed the presence of psychological safety as a theme which emerged in the analysis of qualitative data.

The aims and objectives of all papers were coded and collapsed into themes. The most frequently studied aim relating to psychological safety was “assessing the perceived motivations and barriers to speaking up”. The coding categories for the aims of each study are summarised in Table 2.

**Table 2:** Aims of Included Studies

AIMS OF STUDY	NUMBER OF STUDIES
Assess perceived motivations and barriers to speaking up (one study also looked at how these barriers differed across two cultures) [50–63]	14
Assessment of safety climate / culture / quality / teamwork [64–72]	9
Evaluation of an intervention on speaking up, safety or communication [73–79]	7
Impact of hierarchy on speaking up [80–83]	4
To describe the nature of interprofessional work and the factors that influence teamwork [84–87]	4
Assessment of likely harm and relationship to speaking up [88–91]	4
Impact of management / leadership on psychological safety [21, 92, 93]	3
Explore perceptions of safety following an interprofessional teamwork intervention [94–96]	3
To explore perceptions of own ability to speak up and be heard [97–99]	3
Professional challenges and reasons for wanting to leave [100]	1
Test relationship between speaking up and technical team performance [101]	1
Explore the process of learning to speak up [102]	1
Identify ethical errors in caring for elderly patients with dementia [103]	1
Exploring how the organisation values psychological safety of its workers and makes workers feel valued [104]	1
Identify critical non-technical skills for safe and effective teamwork [105]	1
Assess speaking up behaviour and safety climate [106]	1
Describe advocacy in anaesthesia care [107]	1
Explore experiences of supervision from seniors [108]	1
Observed how staff members spoke up [109]	1
Explore experiences of being a nurse manager [110]	1

### Research objectives

#### Experiences of psychological safety

Each included study was reviewed with a focus upon qualitative data presented within the results (most frequently as interview/focus group transcript excerpts, or open-ended survey responses), and on the conclusions presented within discussion sections.

Data corresponding to experiences of psychological safety or impressions regarding a healthcare worker’s psychological safety were extracted and coded. These codes were organised into a framework including data corresponding to psychological safety, the factors which influenced its presence and associated themes such as error, teamwork and safety.

Data from each study were analysed to gain an overall impression of the psychological safety in each study’s participant group (whether that be “high”, “moderate” or “low”). Sixteen studies demonstrated predominantly low psychological safety (8 qualitative, 5 quantitative and 3 mixed methods). Examples of this included demonstrating nurses not challenging doctors’ practice [82] and that both fear of repercussion and unclear expectations limited an individual’s psychological safety [58]. Only 6 studies (2 qualitative and 4 quantitative) reported a predominance of high psychological safety within their study participants, highlighting that the importance of preventing harm to patients empowered individuals and improved psychological safety [92]. Seven studies demonstrated that they had observed an improvement in psychological safety after an intervention such as interpersonal team training [94]. Fifteen studies did not report homogenous finding for the psychological safety of their participants, with both high and low levels of psychological safety identified within their participant group. The assessment of psychological safety for each study and supporting themes are presented in Additional File 2.

Qualitative data presented within each study in the form of verbatim quotes that related to an individual’s study participants psychological safety were identified and coded into two groups – “high” and “low”.

Examples of low psychological safety highlighted the importance of hierarchy and supportive seniors:

*“a lot of people are still in awe of physicians and will not question physicians”* [88].

*“there is nowhere to turn. They [management] just laugh at you or look through you”* [83]

Data indicating a higher level of psychological safety demonstrated the importance of supportive leadership and shared goals within the team:

*“everyone’s view is listened to, even if it’s in the minority”* [81].

*“we’d done a timeout, we knew each other’s names, we were all focused on the same thing”* [92]

Further examples of data coded into each category are presented in Additional File 3.

The heterogeneity of the data around individual healthcare workers psychological safety across all 62 papers was such that it was not possible to draw an overall or generalisable conclusion about the psychological safety of healthcare workers as a collective. Whilst many of the included studies used quantitative methodology, the wide range of data collection tools and scales prevented an overall compilation of this data, and again assessing the overall presence of psychological safety in this subgroup was not feasible. The data extracted during this thematic analysis were used in the generation of analytical themes, as reported later in this paper.

#### Methods used to assess psychological safety

The 62 included studies utilised a number of different research methodologies, as outlined in Table 3.

**Table 3:** Research methodologies utilised to evaluate psychological safety

**QUALITATIVE METHODOLOGY**	
Interviews [50, 55, 58, 60, 65, 69, 82–84, 91, 92, 96, 98, 100, 102, 105, 107, 108, 110]	19
Focus Groups [94, 97, 103]	3
Simulation (Qualitative Analysis) [109]	1
Ethnographic Observations and Interviews [63]	1
Ethnographic Observations [87]	1
**QUANTITATIVE METHODOLOGY**	
Survey Data [21, 52–54, 56, 57, 59, 61, 62, 66–68, 70–73, 75, 76, 78–81, 85, 86, 88–90, 93, 95, 106]	30
Simulation (Quantitative Analysis) [77]	1
**MIXED METHODS**	
Qualitative Interview and Paired Survey [64, 104]	2
Survey with Qualitative analysis of Open-ended questions [51, 74]	2
Simulation (Qualitative analysis and Quantitative Scoring applied) [99, 101]	2

Within studies using quantitative surveys, 9 used existing survey formats - 3 utilised Edmonson’s safety tool [4], 3 used the safety attitudes questionnaire (SAQ) [111] and 3 used SUPS-Q (Speaking Up about Patient Safety – Questionnaire) [54]. There was an approximate 50:50 split between studies that used qualitative and quantitative methodology with a small proportion (6/62) using mixed methodology. A wide range of qualitative techniques were employed, the most frequent being semi-structured interviews allowing participants to explore their own previous experiences. A review of the available topic guides for studies that used interviews / focus groups showed concordance in the style of questions and topics addressed – including ease in voicing concerns and feelings around speaking up.

It became apparent that there were 4 different approaches to evaluating psychological safety:

1. Participants asked to reflect on their past experiences of psychological safety (*n* = 41)
2. Participants asked to predict how they feel they might behave in hypothetical clinical scenarios (*n* = 8)
3. Evaluation of the change in psychological safety after an intervention (teamworking exercise or structured ward rounds) (*n* = 9)
4. Participants observed during simulated scenarios as a technique to explore their psychological safety. (*n* = 4)

#### Evidence of consequences of high or low psychological safety

Eighteen of the included studies either investigated outcomes associated with psychological safety, or inferred consequences as a result of the climate of psychological safety identified.

Within this subgroup were 9 quantitative studies using surveys, 8 qualitative studies (6 utilised interviews and 2 used focus group) and 1 study using simulation and subsequent quantitative scoring of observed interactions.

Nine studies found a statistically significant relationship between the presence of psychological safety and a defined outcome measure – with high psychological safety positively related to creative performance and knowledge sharing [57], technical team performance [101], improving continuous quality improvement [70] and patient centred care [70]. Psychological safety was positively associated with learning from failure and performance [81]. Low psychological safety was negatively correlated with speaking up and withholding voice [54]. The remaining studies in this subgroup used data reported by participants about their experience in the clinical environment to infer correlations and associations between psychological safety and possible consequences. The most commonly inferred association by study authors was that low psychological safety had a negative impact on patient risk of harm, identified in 10 studies. Data extracted during the content analysis can be viewed in Additional File 4.

#### Analytical themes

The thematic analysis led to the development of two themes which go beyond the content of the original studies. These themes were the presence of facilitators and barriers to psychological safety, and the influence of situational context on the psychological safety of healthcare workers.

Data extraction and coding of descriptive items captured within the thematic analysis provided detailed information regarding the facilitators and barriers to psychological safety within the workplace, as perceived by each individual participant. Discussion and analysis within the research team allowed this data to be organised into higher order categories. These categories included culture, workload, infrastructure, teamwork and motivation. These categories and data within each category relating to facilitators and barriers of psychological safety are demonstrated in Additional File 5.

**Fig. 2:**
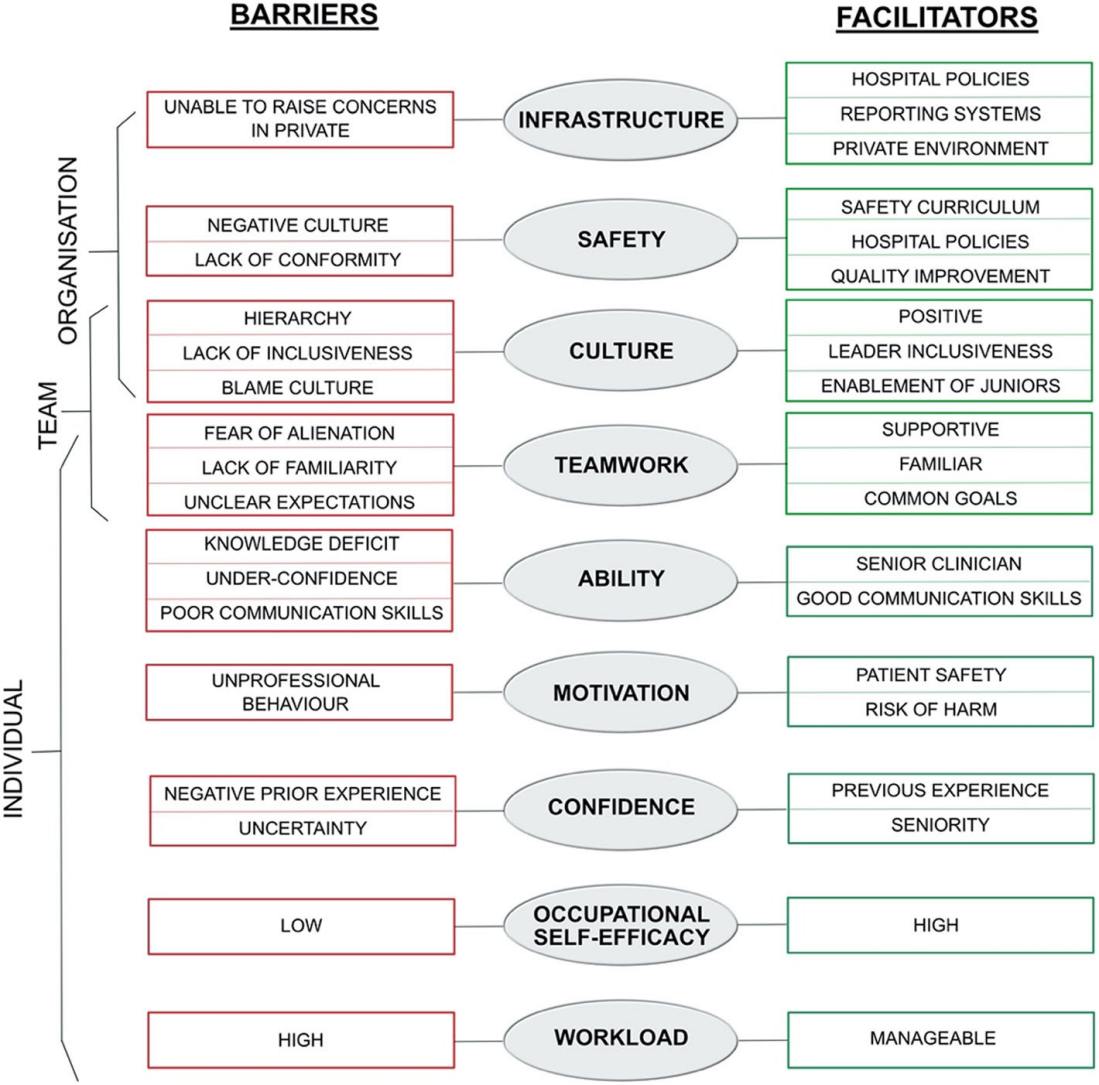
Diagram illustrating the barriers and facilitators to psychological safety and where they were significant within the workplace

These categories were organised into a framework outlining the level within the workplace at which they were significant – individual, team or organisational (it was possible for a factor to be relevant at more than one level). This concept is illustrated in Fig. 2.

The second analytical theme that emerged was surrounding the influence of situational context (as associated with every clinical scenario) on psychological safety. The influence of situational context (defined as “variables that influence or could influence the ‘independent’ and dependent variable directly under study” [112]) on psychological safety emerged as a recurrent theme within the thematic analysis. The analysis evolved into the development of a framework and subsequently a model outlining the dynamics associated with situational context and their potential impact on psychological safety and outcomes within the healthcare environment.

This thematic analysis had 3 stages. First the data were re-reviewed for evidence relating to situational context as defined. Subsequently the relationship of this data to the presence of psychological safety was explored. Thirdly we analysed how situational context might influence the healthcare worker or clinical situation through its effect on psychological safety.

**Fig. 3:**
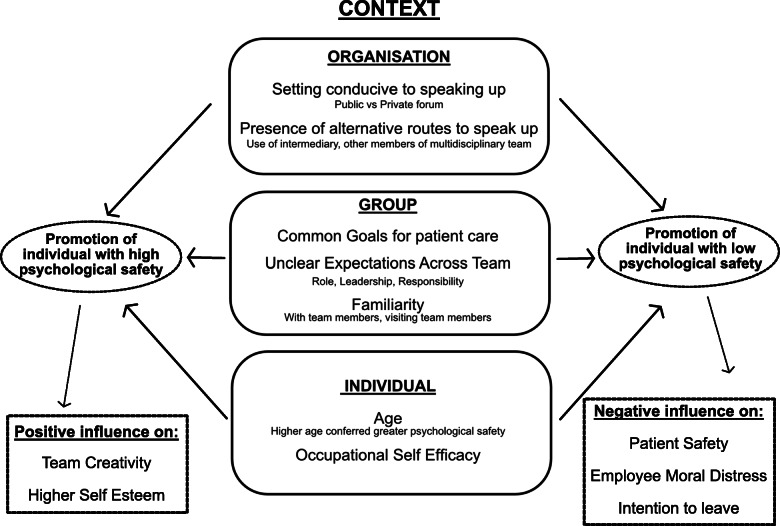
A model illustrating the influence of situational context on psychological safety within the healthcare environment

Data within our model were again organised within a framework identifying the level within the workplace that each aspect of situational context was relevant (Fig. 3). The figure highlights some of the contextual factors at each level that are not frequently considered in other models of psychological safety, and their particular relevance to a healthcare setting.

#### Quality appraisal

Twenty-eight of the included studies used a purposive sampling technique. This is a well utilised sampling technique within qualitative research and provides a representation of the sample population studied (rather than being generalisable across a wider population). It is possible that within each study population there are individuals who were not represented. It is also impossible to generalise the findings of one paper to the entire population of healthcare workers.

However, sampling for proportionality was not the main concern within these studies, therefore a potential bias in recruitment is unlikely to be detrimental to the overall conclusions of each study and this evidence synthesis.

Studies that were wholly qualitative in nature were less susceptible to selection bias than those who applied a stratified sampling technique (for example those who distributed surveys to entire departments and relied on individuals motivated to participate), given the fact they recruited until thematic saturation was achieved.

Given the desired outcomes of each study, and of this evidence synthesis, no study was rejected due to risk of bias or methodological limitations. Qualitative studies were not excluded on the basis of our assessment of their methodological limitations, but this information was used in the assessment of our confidence in the synthesis findings.

## Discussion

This evidence synthesis collates a broad range of worldwide data to provide information regarding psychological safety in healthcare workers, as well as being the largest collation of papers exploring the topic of psychological safety to date. This study found that there was substantial variation in the psychological safety reported by healthcare workers across all studies, and evidence that there is an ongoing need to focus upon its improvement. We highlight a huge variety in the methods used to evaluate psychological safety within the literature and demonstrate that there is evidence that the presence of psychological safety has an impact on the clinical environment, both for the healthcare workers themselves and their patients. The thematic analysis undertaken to address our original research objectives yielded two themes which go beyond the content of the included studies and provide a novel contribution to the existing literature.

Individuals possessing high levels of psychological safety are crucial to effective and safe healthcare delivery, and also in the promotion of organisational learning. Such individuals contribute by discussing risk and adapting to avoid error; consequently, the organisation can find new pathways and processes to facilitate future positive outcomes. This evidence synthesis corroborates existing themes (such as the importance of leader inclusiveness [19]) and builds on models created by previous reviews [19, 113–115] about the relationships contributing to psychological safety at the individual, team and organisational level.

The data synthesised in this study is reassuring for healthcare leaders – we demonstrate that psychological safety is consistently shown to be present (often to high levels) within the populations of healthcare workers studied. However, the analysis demonstrated that there is consistently a number of individuals who report feelings and behaviours consistent with low psychological safety.

There was little consistency in the methods used to assess psychological safety. A large number of studies incorporated the use of quantitative surveys, but these were often developed by the authors themselves, as opposed to drawing on tools already available. As such there is scope to validate an existing assessment tool for psychological safety in the population of healthcare workers.

Several studies indicated that psychological safety had a significant benefit on the working environment, particularly when applied to teamwork, team creativity and quality improvement. Whilst no studies provided statistically significant evidence of correlations between low psychological safety and adverse outcomes (impossible in the case of the qualitative studies) there was a strong feeling that this had a negative impact on patient safety. This was predictable based upon prior research; but it was also interesting to observe some of the potential consequences for the individual worker – low self-esteem, increased intention to leave the profession and risk of moral distress. No studies were designed with this outcome in mind, and as such these are opinion at best, but the signal that low psychological safety is detrimental both to the service delivered and the individual healthcare worker is present and warrants further investigation.

A very clear theme regarding the facilitators and barriers to psychological safety experienced by individual healthcare workers worldwide emerged during this thematic analysis. Many of these barriers and facilitators are evidenced in existing literature – such as an individual’s confidence, supportive senior staff and management, feedback from previous episodes of speaking up and the presence of a strong hierarchy [21]. Barriers to speaking up less commonly acknowledged within the existing literature included the influence of an individual’s current workload, the reason itself for speaking up (patient safety was a big motivator, however if related to unprofessional behaviour individuals appeared less likely to speak up) and fear of conflict in front of patients. It is not surprising that effective reporting channels, all members of the team feeling enabled and high occupational self-efficacy were key in promoting psychological safety. These are examples of both organisational factors (the improvement of reporting channels and feedback to individuals), and individual factors – improving confidence and knowledge base.

We contribute to the existing literature by providing a detailed explanation about how situational context can influence psychological safety within the healthcare sector. We illustrate how the factors that contribute to this context are present at all levels – individual, team and organisation. This context can be precarious and will change depending upon the factors associated with each clinical event. For example, at the organisational level, within the category of infrastructure, we observed that the setting for speaking up had an impact on an individual’s psychological safety. An individual may feel confident to speak up in the context of a private setting but be constrained by the context of a public venue.

Organisational culture has an influence on situational context and the consequential perception of a psychologically safe environment. Changing the culture of an organisation, envisaged with a view to improving psychological safety, can be challenging due to the presence of multiple stakeholders and the complexities associated with the healthcare setting [14]. Team leaders have a crucial role in mitigating these challenges and promoting a psychologically safe environment through leadership behaviours such as inclusiveness and being change oriented [16]. Our model demonstrating the influence of situational context provides a framework for team leaders wanting to implement change that allows the understanding and subsequent management of the dynamics associated with situational context within the healthcare environment. The model also highlights how context can impact the perception of psychological safety by an individual, with certain contextual factors promoting either high or low psychological safety. In practice this understanding of context is related to a leader’s situational awareness [116]. If leaders understand where scenarios leading to low psychological safety may arise, they can use this framework to perceive potential issues within the workplace, identify them as relevant and project the potential impact. Through this, leaders may be able to modify the situational context and subsequently improve psychological safety.

There are limitations within this evidence synthesis. Whilst we intentionally kept the search strategy broad there is still the possibility that some qualitative papers did not appear within our search as a consequence of unreliable indexing. However, this is unlikely to have had a significant impact on the findings of this thematic analysis, as the large number of papers included allowed a point of thematic saturation to be achieved, in line with qualitative methodology [117, 118].

We have addressed the possibility of selection bias within individual papers, or that entire populations may not be accurately represented (particularly in relation to quantitative surveys with lower response rates). Given the aim of this study was to explore psychological safety, the themes and concepts drawn from the data are still extremely useful, even if some surveys had the potential for a negative selection bias (i.e., only those who had conflict / dissatisfaction to report volunteered to participate). It is also possible that centres participating in studies investigating teamwork & safety were more aligned to these ideals in the first place, thereby introducing the possibly of some sampling bias.

Another consideration is the approach used to assess psychological safety. Forty-one papers required individuals to reflect on their previous behaviour – which assumes that their recollection is reliable. This also raises the question – is asking people to predict how they might behave an accurate reflection of real-life behaviour, given that people are unlikely to volunteer that they would behave in a way that may be detrimental to patient safety.

Several opportunities for future research are highlighted in this evidence synthesis. Firstly, there is scope to validate a tool specifically for the assessment of psychological safety within healthcare workers.

Many of the studies looking at psychological safety within the population of healthcare workers focus on how it relates to voicing concerns and the subsequent benefits for patient safety. This is one manifestation of a psychologically safe environment; however, it is important to explore other expressions of psychological safety such as innovation and organisational commitment [5], and to explore for links to staff satisfaction and career longevity.

This study is limited to exploring the presence of psychological safety within study participants and does not look at what may be contributing to different levels in different countries. It would be interesting to further explore how cultural factors may influence psychological safety, particularly within the context of a multi-cultural workplace environment.

At best this evidence synthesis highlights areas where situational context may influence an individual’s psychological safety and the variables which may be affected by this (such as patient safety). Further research is required, perhaps in the form of an ethnographic study to observe the impact of situational context on psychological safety and further analyse the determinant role of situational context on psychological safety.

## Conclusion

This evidence synthesis provides positive data regarding the presence of psychological safety within healthcare workers, whilst illuminating areas for improvement. We add more detail to the current literature regarding the facilitators and barriers of psychological safety and highlight how situational context can influence the creation of a psychologically safe environment.

There are many factors which can oppose the presence of psychological safety – including the influence of the team leader, personalities of individual team workers, the responsibility associated with the decisions required of the team and the speed at which decisions need to be made. These are likely to be consistent across most healthcare environments, and as such the findings of this evidence synthesis are transferrable across different clinical environments and populations of healthcare workers.

Many of the factors that contribute to psychological safety are not malleable or easy to change (especially within the constraints of a resource poor environment). It is also likely to be the case that some factors promoting psychological safety will be unique to the team itself, and the individual personalities and stresses that are found within that particular environment [119, 120]. Through improved understanding of the contributing factors to psychological safety and the areas in which situational context is especially important it is possible that some modifiable factors will be identified. This information can be used by team leaders and management to promote psychological safety within their clinical environment.

## Supplementary Information

**Additional file 1:** Summary of Study Characteristics Table.

**Additional file 2:** Results of Individual Studies.

**Additional file 3:** Supporting Quotes: Experiences of Psychological Safety.

**Additional file 4:** Possible consequences of high or low psychological safety.

**Additional file 5:** Facilitators and Barriers to Psychological Safety.

## Data Availability

The datasets used and/or analysed during the current study are available from the corresponding author on reasonable request.
