# Supplementary material for: The presence and potential impact of psychological safety in the healthcare setting: an evidence synthesis
Source: BMC Health Serv Res. 2021 Aug 5;21:773. doi: 10.1186/s12913-021-06740-6 (PMC8344175; doi:10.1186/s12913-021-06740-6)
Supplement: Supplementary file 1 — Additional file 1: Summary of Study Characteristics Table. [file 12913_2021_6740_MOESM1_ESM.docx]

**ADDITIONAL FILE 1:**

**Summary of Study Characteristics Table**

| **Study** | **Location: Country, Site Type** | **Clinical Department** | **Sample of Healthcare workers (n)** | **Method of Sampling Participants** | **Study Outcome Measures** | **Tools Used to Assess Psychological Safety** | **Research Methodology** | **Psychological Safety as an Outcome Measure** |
| --- | --- | --- | --- | --- | --- | --- | --- | --- |
| Abdi et al, 2015 (40) | Iran, Hospital | Intensive Care | Nurses and Physicians (42) | Purposive Sampling Technique | Assessment of Safety Climate within ICU | Qualitative Interviews and Survey Data (SAQ) | Mixed Methods | Theme that emerged during analysis |
| Alilu et al, 2014 (77) | Iran, Hospital | Multiple Clinical Areas | Nurses  (16) | Purposive Sampling Technique | To explore professional challenges in clinical settings and reasons for wanting to leave the profession | Qualitative Interviews - Content Analysis of Transcripts | Qualitative | Theme that emerged during analysis |
| Alingh et al, 2014  (70) | Netherlands, Hospital | Multiple Clinical Areas | Nurses and Nurse Managers  (1073) | Stratified Sampling Technique | To explore the relationships between control-based and commitment-based safety management, climate for safety, psychological safety and nurses’ willingness to speak up | Cross Sectional Survey Study | Quantitative - Survey Data | Primary Objective |
| Attree et al, 2006 (26) | United Kingdom, Hospital | Multiple Clinical Areas | Nurses  (142) | Purposive Sampling Technique | To explore factors that influence nurses’ decisions to raise concerns about standards of practice. | Qualitative Interviews - Grounded Theory Approach | Qualitative | Primary Objective |
| Aveling et al, 2015 (41) | East Africa, Hospital | Multiple Clinical Areas | Hospital Staff - all areas  (57) | Purposive Sampling Technique | Explore how healthcare workers in two East African hospitals identify and explain the major obstacles to ensuring the safety of patients in their care. | Qualitative Interviews - Thematic Analysis | Qualitative | Theme that emerged during analysis |
| Baik et al, 2016 (71) | USA, Hospital | Cardiac Unit | Nurses  (10) | Convenience Sampling Technique | Explore nurses’ experiences and perceptions following a purposeful interprofessional team intervention in practice | Focus Groups - analysis of transcripts | Qualitative | Theme that emerged during analysis |
| Belyansky et al, 2010 (56) | USA, Hospital | Surgery | Physicians  (72) | Stratified Sampling Technique | Does surgical hierarchy interfere with resident voicing concerns about patient safety | Cross Sectional Survey Study | Quantitative - Survey Data | Primary Objective |
| Churchman et al, 2008 (58) | United Kingdom, Hospital | Multiple Clinical Areas | Nurses  (12) | Purposive Sampling Technique | To explore the extent to which nurses are willing to challenge doctors’ practice in everyday situations in an acute NHS hospital | Qualitative Interviews - Thematic Analysis | Qualitative | Primary Objective |
| Edrees et al, 2012 (27) | United Arab Emirates, Hospital | Intensive Care | ICU Staff  (369) | Stratified Sampling Technique | To assess perceived barriers to speaking up and to provide recommendation for reducing barriers to reporting adverse events and near misses | Survey with thematic analysis of open-ended questions | Mixed Methods | Primary Objective |
| Etchegaray et al, 2010 (49) | USA, Hospital | Multiple Clinical Areas | Hospital Staff with direct patient care - all areas  (2933) | Stratified Sampling Technique | Creation of a reliable and valid high-performance work system (HPWS) survey and evaluation of this, culture and outcomes | Survey | Quantitative - Survey Data | Primary Objective |
| Farag et al, 2014 (28) | USA, Hospital | Critical and Acute Care | Nurses  (144) | Stratified Sampling Technique | To assess safety motivation - willingness of nurses to report error with 2 aims | Cross Sectional Survey Study | Quantitative - Survey Data | Primary Objective |
| Garon et al, 2009 (74) | USA, Hospital | Multiple Clinical Areas | Nurses and Nurse Managers  (33) | Purposive Sampling Technique | To explore nurses’ perceptions of their own ability to speak up and be heard in the workforce | Focus Groups - analysis of transcripts | Qualitative | Primary Objective |
| Garon et al, 1998, 2004 (75) | USA, Hospital | Multiple Clinical Areas | Nurses  (19) | Purposive Sampling Technique | To relate nurses’ stories of their experiences of acts of resistance | Qualitative Interviews - Narrative Analysis | Qualitative | Primary Objective |
| Gauld et al, 2012 (42) | New Zealand, Hospital | Multiple Clinical Areas | Hospital Staff - all areas  (10,303) | Stratified Sampling Technique | Measure health professional perceptions of quality and safety across an entire system of public hospitals. | Survey | Quantitative - Survey Data | Primary Objective |
| Gausvik et al, 2014 (50) | USA, Hospital | Elderly Care | Nurses and Allied Healthcare Professionals  (24) | Stratified Sampling Technique | To measure percep­tions of teamwork, communication, understanding of the plan for the day, safety, efficiency, and job satisfaction | Survey with thematic analysis of open-ended questions | Mixed Methods | Theme that emerged during analysis |
| Hemingway et al, 2006 (51) | USA, Hospital | Surgery | Theatre Staff  (169) | Stratified Sampling Technique | Assess outcome of process change in the following areas: Safety reporting measures, safety debriefings, audits, communication methods | Survey | Quantitative - Survey Data | Theme that emerged during analysis |
| Hirak et al, 2011 (57) | Isreal, Hospital | Multiple Clinical Areas | Nurses and Physicians  (277) | Random Sampling Technique | Analyse how the behaviour of the leader influences psychological safety and learning from failure | Survey | Quantitative - Survey Data | Primary Objective |
| Hughes et al, 2014 (52) | USA, Hospital | Emergency Department | Trauma Team Members  (132) | Stratified Sampling Technique | Explore changes after implementation of a crew resource mangement programme | Survey | Quantitative - Survey Data | Primary Objective |
| Jayasuriya-Illesinghe et al, 2016 (60) | Sri Lanka, Hospital | Surgery | Surgeons  (15) | Purposive Sampling Technique | To describe the nature of interprofessional work and the factors that influence teamwork in this setting | Qualitative Interviews - Thematic Analysis | Qualitative | Secondary Objective |
| Kaafarani et al, 2006 (43) | USA, Hospital | Theatre and Recovery | Theatre and Recovery Staff  (324) | Stratified Sampling Technique | To evaluate patient safety culture in the OR and PACU | Survey | Quantitative - Survey Data | Secondary Objective |
| Kessel et al, 2012 (33) | Germany, Mixed Primary and Secondary | Multiple Clinical Areas | Healthcare teams caring for patients with rare diseases  (149) | Stratified Sampling Technique | How does Psychological safety foster knowledge-sharing process and enable team creative performance? | Survey - Edmondson’s Tool | Quantitative - Survey Data | Primary Objective |
| Kobayashi et al, 2005 (32) | USA, Japan. Hospitals | Multiple Clinical Areas | Physicians  (240) | Stratified Sampling Technique | To identify perceived barriers to residents’ questioning or challenging their seniors, to determine how these barriers affect decisions and to assess how these barriers differ across cultures | Survey | Quantitative - Survey Data | Primary Objective |
| Kolbe et al, 2012 (78) | Switzerland, Hospitals | Simulator | Anaesthesia Residents and Nurses  (62) | Stratified Sampling Technique | To test the relationship between speaking up and technical team performance in anaesthesia | Scored Simulated Encounters | Mixed Methods | Primary Objective |
| Law et al, 2014 (79) | China, Hospitals | Multiple Clinical Areas | Nurses  (18) | Purposive Sampling Technique | To explore the process of learning to speak up in practice among newly graduated registered nurses | Qualitative Interviews - Iterative Analysis | Qualitative | Primary Objective |
| Livorsi et al, 2014 (34) | USA, Hospital | Intensive Care | ICU Staff  (39) | Purposive Sampling Technique | To explore three safety domains: reporting errors, approachability of authority figures and handovers. | Qualitative Interviews - Thematic Analysis | Qualitative | Primary Objective |
| Lockett et al, 2015 (45) | USA, Hospital | Multiple Clinical Areas | Nurses and Nurse Managers  (28) | Purposive Sampling Technique | To define and create a conceptual model for peer-to-peer accountability | Qualitative Interviews - Grounded Theory Approach | Qualitative | Primary Objective |
| Lyndon et al, 2012 (64) | USA, Hospital | Labour and Delivery Unit | Nurses and Obstetricians  (125) | Stratified Sampling Technique | Assessment of likely harm and relationship to speaking up | Survey | Quantitative - Survey Data | Primary Objective |
| Malloy et al, 2009 (80) | Canada, Korea, Australia, Ireland. Hospital | Elderly Care | Nurses  (42) | Purposive Sampling Technique | Identify ethical dilemmas in caring for elderly people with dementia | Focus Groups - analysis of transcripts | Qualitative | Theme that emerged during analysis |
| Martinez et al, 2014 (29) | USA, Hospital | Multiple Clinical Areas | Interns and Residents  (1800) | Stratified Sampling Technique | To compare interns’ and residents’ experiences, attitudes and factors associated with speaking up about traditional vs professionalism related safety threats | Survey | Quantitative - Survey Data | Primary Objective |
| Maxfield et al, 2013 (65) | USA, Hospital | Labour and Delivery Unit | Physicians, Midwives and Nurses  (3282) | Stratified Sampling Technique | To assess the occurrence of 4 safety concerns among labor and delivery teams: dangerous shortcuts, missing competencies, disrespect and performance problems. | Survey | Quantitative - Survey Data | Secondary Objective |
| McLinton et al, 2017 (81) | Australia, Hospital | Multiple Clinical Areas | Hospital Staff - all areas  (27) | Purposive Sampling Technique | To explore key themes: “whether the organisation valued the psychological safety of the workers as well as physical, and how management practices make workers feel valued | Qualitative Interviews with grounded theory approach to analysis, subsequently linked to survey data | Mixed Methods | Primary Objective |
| Nembhard et al, 2005 (68) | USA, Hospital | Neonatal Intensive Care Unit | NICU Staff  (23) | Purposive Sampling Technique | To test the hypothesis that leader inclusiveness promotes psychological safety | Survey - Edmondson’s Tool | Quantitative - Survey Data | Primary Objective |
| Ortega et al, 2011 (44) | Spain, Hospital | Multiple Clinical Areas | Hospital Staff - all areas  (468) | Stratified Sampling Technique | Explore the relationship between team level learning and performance in nursing teams – and the role of beliefs about interpersonal context in this relationship | Cross Sectional Survey Study | Quantitative - Survey Data | Primary Objective |
| Pian-Smith, 2009 (53) | USA, Hospital | Simulator | Anaesthesia Residents  (44) | Purposive Sampling Technique | Evaluate the effect of teaching residents the two-challenge rule | Scored Simulated Encounters | Quantitative | Primary Objective |
| Piers et al, 2017 (61) | Belgium, Hospital | Elderly Care | Nurses and Allied Healthcare Professionals  (890) | Stratified Sampling Technique | To explore the quality of interprofessional teamwork in acute geriatric care and to build a model of team types | Cross Sectional Survey Study | Quantitative - Survey Data | Theme that emerged during analysis |
| Putnam et al, 2015 (72) | USA, Hospital | Surgery | Theatre Staff  (63) | Stratified Sampling Technique | To test the effect of an enhanced safety curriculum | Survey - SAQ | Quantitative - Survey Data | Primary Objective |
| Raemer et al, 2016 (86) | USA, Hospital | Simulator | Anaesthesia Residents  (40) | Stratified Sampling Technique | Three questions: -Would an educational intervention be effective in improving speaking-up behaviours of practicing non-trainee anaesthetists when presented with realistic simulated clinical situations? -What would those speaking up behaviours be? -What were the hurdles and enablers? | Analysis Simulated encounters | Qualitative - coding of simulated encounters | Primary Objective |
| Rathert et al, 2009 (46) | USA, Hospital | Acute Care | Hospital Staff - all acute areas  (306) | Stratified Sampling Technique | To empirically explore a theoretical model linking the work environment in the healthcare setting and how it might relate to work engagement, organisational commitment and patient safety | Survey - Edmondson’s Tool | Quantitative - Survey Data | Primary Objective |
| Richard et al, 2017 (30) | Switzerland, Hospitals | Paediatrics | Nurses and Physicians  (523) | Stratified Sampling Technique | To develop a short questionnaire allowing healthcare organisations to assess different aspects of speaking up among healthcare staff | Survey | Quantitative - Survey Data | Primary Objective |
| Roberts et al, 2014 (31) | USA, Hospital | Paediatrics - General medical and surgical care | Nurses and Physicians  (57) | Purposive Sampling Technique | To proactively identify barriers to calling for urgent assistance that exist despite recent implementation of a comprehensive RRS in a children’s hospital | Qualitative Interviews - Modified Grounded Theory Approach | Qualitative | Primary Objective |
| Roussin et al, 2018 (35) | Spain, Hospital | Simulator | Nurses and Physicians  (129) | Stratified Sampling Technique | Test the assumption that greater feelings of self-efficacy and psychological safety would be associated with greater speaking up in simulation-based healthcare learning environments | Survey | Quantitative - Survey Data | Primary Objective |
| Rutherford et al, 2012 (82) | United Kingdom, Hospital | Theatre and Recovery | Anaesthetic Consultants and Assistants  (33) | Purposive Sampling Technique | To identify the critical non-technical skills for safe and effective anaesthetic assistant performance | Qualitative Interviews - Thematic Analysis | Qualitative | Primary Objective |
| Sayre et al, 2012 (54) | USA, Hospital | Acute Care | Nurses  (104) | Purposive Sampling Technique | Whether in-service training could lead nurses to speak up, thereby enhancing perceptions of collaboration | Survey - "Speaking Up Measure" | Quantitative - Quasi-experimental research design | Primary Objective |
| Schwappach and Gering, 2014 (36) | Switzerland, Hospitals | Oncology (Adult and Paediatric) | Oncology Nurses and Physicians  (32) | Purposive Sampling Technique | To investigate the motivations and barriers to speaking up towards co-workers and supervisors | Qualitative Interviews - Inductive thematic content analysis | Qualitative | Primary Objective |
| Schwappach and Gering, 2014 (37) | Switzerland, Hospitals | Oncology (Adult and Paediatric) | Oncology Nurses and Physicians  (1013) | Stratified Sampling Technique | To investigate the likelihood of speaking up about patient safety in oncology and to clarify the effect of clinical and situational context factors on the likelihood of voicing concerns | Survey | Quantitative - Survey Data | Primary Objective |
| Schwappach and Richard, 2018 (83) | Switzerland, Hospitals | Multiple Clinical Areas | Nurses and Physicians  (979) | Stratified Sampling Technique | To determine frequencies of healthcare workers speak up-related behaviours and the association of speak up-related safety climate with speaking up and withholding voice | Survey | Quantitative - Survey Data | Primary Objective |
| Schwappach and Sendlhofer (47) | Switzerland, Austria. Hospitals | Multiple Clinical Areas | Staff working in surgical and anaesthesia department  (768) | Stratified Sampling Technique | To compare speaking up related climate and behaviours in academic and non-academic hospitals. | Survey - SUPS-Q | Quantitative - Survey Data | Primary Objective |
| Schwappach et al, 2018 (66) | Switzerland, Hospitals | Multiple Clinical Areas | Nurses and Physicians  (1217) | Stratified Sampling Technique | To investigate speaking up behaviours, safety climate and likelihood to speak up about poor hand hygiene practice descripted in a vignette | Cross Sectional Survey Study - SUPS-Q | Quantitative - Survey Data | Primary Objective |
| Schwappach and Sendlhofer, 2018 (48) | Austria, Hospital | Multiple Clinical Areas | Hospital Staff - all acute areas  (859) | Stratified Sampling Technique | To analyse speaking up behaviour and safety climate with a validated questionnaire for the first time in an Austrian university hospital | Survey | Quantitative - Survey Data | Primary Objective |
| Sexton et al, 2006 (62) | USA, Hospital | Surgery | Theatre Staff  (2135) | Stratified Sampling Technique | To test the psychometric properties of a teamwork climate scale in the OR setting, and to provide some initial benchmarking information on OR teamwork climate by hospital and caregiver type | Survey - SAQ | Quantitative - Survey Data | Primary Objective |
| Sundqvist et al, 2013 (84) | Sweden, Hospital | Anaesthesia | Nurse Anaesthetists  (20) | Purposive Sampling Technique | The aim of the study was to describe advocacy in anaesthesia care during the perioperative phase from the perspective of the registered nurse anaesthetist | Qualitative Interviews - Content Analysis of Transcripts | Qualitative | Theme that emerged during analysis |
| Sur et al, 2015 (69) | USA, Hospital | Surgery | Surgical Residents  (18) | Purposive Sampling Technique | To evaluate trainees approaches to concerns about supervisor’s clinical decision making | Qualitative Interviews - Thematic Analysis | Qualitative | Primary Objective |
| Szymczak et al, 2015 (67) | USA, Hospital | Paediatrics | Physicians  (103) | Purposive Sampling Technique | To examine how clinicians talk about speaking up or not in the face of breaches in infection prevention technique | Qualitative Interviews - Thematic Analysis | Qualitative | Primary Objective |
| Tamuz et al, 2011 (85) | USA, Hospital | Intensive Care | Physicians  (17) | Purposive Sampling Technique | To explore how residents in the ICU experienced supervision related to medication safety, not only from supervising physicians but also from other professionals | Qualitative Interviews - Thematic Analysis | Qualitative | Theme that emerged during analysis |
| Tangirala et al, 2008 (38) | USA, Hospital | Multiple Clinical Areas | Nurses  (586) | Stratified Sampling Technique | To investigate the relationship between personal control (employees’ perceptions of autonomy and impact at work) and voice (employees’ expression of challenging but constructive work-related opinions, concerns or ideas | Survey | Quantitative - Survey Data | Primary Objective |
| Tarrant et al, 2017 (39) | United Kingdom, Hospital | Intensive Care | ICU Staff  (98) | Purposive Sampling Technique | To study how personnel gave voice to concerns about patient safety or poor practice | Ethnographic observations and qualitative interviews - Thematic analysis | Qualitative | Primary Objective |
| Todorova et al, 2012 (59) | Bulgaria, Hospital | Multiple Clinical Areas | Nurses and Physicians  (42) | Purposive Sampling Technique | To explore health professionals’ perceptions of organisational hierarchies and how doctors and nurses connect these to organisational justice | Qualitative Interviews - Thematic Analysis | Qualitative | Theme that emerged during analysis |
| Urisman et al, 2018 (55) | USA, Hospital | Intensive Care | ICU Staff  (65) | Stratified Sampling Technique | To evaluate the impact of introducing an interdisciplinary rounding format that formalised the participation of nurses in rounds on the effectiveness of interprofessional collaboration, specifically between nurses and physicians and (ii) to evaluate a possible impact of adopting these rounds on measurable patient outcomes | Survey | Quantitative - Survey Data | Theme that emerged during analysis |
| Van Bogaert et al, 2015 (87) | Belgium, Hospital | Multiple Clinical Areas | Medical or Surgical Nurse Managers  (8) | Purposive Sampling Technique | To study nurse managers’ perceptions and experiences of staff nurse structural empowerment and its impact on the nurse manager leadership role and style | Qualitative Interviews - Thematic Analysis | Qualitative | Theme that emerged during analysis |
| Weiss et al, 2014 (76) | Switzerland, Hospitals | Simulator | Anaesthesia Physicians and Nurses  (27) | Purposive Sampling Technique | To investigate individual predictors of speaking up in acute care teams | Scored Simulated Encounters and Survey | Mixed Methods | Primary Objective |
| Weller et al, 2011 (73) | New Zealand, Primary and Secondary Care | Multiple Clinical Areas | Junior Doctors and Nurses  (25) | Purposive Sampling Technique | To understand the nature of the interactions, activities and issues affecting new medical and nursing graduates in order to inform interventions to improve IPC among junior health professions graduates | Qualitative Interviews - Thematic Analysis | Qualitative | Theme that emerged during analysis |
| Whitehair et al, 2018 (63) | Australia, Hospital | Paediatrics | Nurses  (7) | Purposive Sampling Technique | To explore how team processes support nursing teams in hospital units during everyday work | Ethnographic observations - Thematic Analysis | Qualitative | Theme that emerged during analysis |
