# Supplementary material for: The presence and potential impact of psychological safety in the healthcare setting: an evidence synthesis
Source: BMC Health Serv Res. 2021 Aug 5;21:773. doi: 10.1186/s12913-021-06740-6 (PMC8344175; doi:10.1186/s12913-021-06740-6)
Supplement: Supplementary file 2 — Additional file 2: Results of Individual Studies. [file 12913_2021_6740_MOESM2_ESM.docx]

**ADDITIONAL FILE 2:**

**Results of Individual Studies**

| **Study** | **Level of Psychological Safety Identified** | **Themes Supporting Level of Psychological Safety** | **Evidence of Consequences of Psychological Safety** |
| --- | --- | --- | --- |
| Abdi et al, 2015 (40) | Generally low - low safety climate, teamwork and job satisfaction scores. | Nursing staff felt discouraged from reporting error due to hierarchy, Nursing staff reported it difficult to express opposing views, Dependent upon individuals involved in each interaction. | N/A |
| Alilu et al, 2014 (77) | Low | Blame assigned by management following errors/complaints, Poor support by management. Example: “humiliation harassment and inappropriate treatment by physicians” | Increased intention to leave profession |
| Alingh et al, 2014 (70) | Study does not quantify overall level but investigates association between psychological safety and other factors. | Psychological safety positively related to speaking up intentions | N/A |
| Attree et al, 2006 (26) | Both high and low levels of psychological safety reported. Felt to be "High risk, low benefit" | Barriers: fear of repercussions, retribution and blame. Perceptions of a closed, concealing and blaming organisation culture. Facilitating factors: Open culture, perception of raising concerns as professional duty. | N/A |
| Aveling et al, 2015 (41) | Low | "Poor teamwork due to hierarchical dynamics". Staff who were not physicians feeling disrespected. "Failure to act upon concerns raised by colleagues" | Perception of undesirable consequences for patient safety |
| Baik et al, 2016 (71) | Improved post intervention (Team building exercise) | Intervention enabled nurses to be an active member of the team. The focus on creating a psychologically safe environment post intervention encouraged them to bring up concerns and issues about patients | N/A |
| Belyansky et al, 2010 (56) | Moderate | Large proportion of participants had seen an adverse event prevented by speaking up. Attendings felt that they encouraged speaking up and welcomed feedback. Not reflected in data from residents | Perception of undesirable consequences for patient safety |
| Churchman et al, 2008 (58) | Low | Lots of examples of nurses who did not challenge doctors’ practice. Assisted by policies or hospital documents. | N/A |
| Edrees et al, 2012 (27) | Statements inferring low levels of psychological safety within paper | Perceptions of a culture of blame and issues with reporting procedures | N/A |
| Etchegara et al, 2010 (49) | Generally high (Agree or strongly agree on Likert scale) | Found that HPWS were a strong predictor of speaking up – better than other tools used | N/A |
| Farag et al, 2014 (28) | Moderate - high scores reflecting willingness to speak up | Authors question scores felt that true score was likely to be lower - “possibility of social desirability associated with study outcome” | N/A |
| Garon et al, 2009 (74) | Both high and low levels of psychological safety reported. | Idea that ability to speak up is shaped by personal experiences and influences at home which are then carried into the workplace. Outcomes: staff want to know the issue they bring up is acted upon. If no response seen deemed pointless to speak up | Perception of undesirable consequences for patient safety |
| Garon et al, 1998, 2004 (75) | Both high and low levels of psychological safety reported. | All participants had examples of speaking up. Difficulty reported with hierarchy, but improved with experiences | Perception of undesirable consequences for patient safety |
| Gauld et al, 2012 (42) | Moderate | Nurses more likely to speak up than other allied health professionals, Older staff more likely to speak up than younger. | N/A |
| Gausvik et al, 2014 (50) | Improved post intervention (Team building exercise) | Patient safety large motivation to speak up: "I feel like I am part of a team dynamic that is really invested in the patient" | N/A |
| Hemingway et al, 2006 (51) | Improved post intervention (Team building exercise) | Patient safety main motivator to speak up | N/A |
| Hirak et al, 2011 (57) | High scores on survey for psychological safety. | Psychological safety positively related to leader inclusiveness, good unit culture | Suggestion that poor performance leads to negative repercussions and thereby reducing psychological safety. |
| Hughes et al, 2014 (52) | Low, even despite improvements post intervention. | Improvement in domain: "Staff are afraid to ask questions when something does not seem right" | N/A |
| Jayasuriya-Illesinghe et al, 2016 (60) | Low, Clear examples of observed low psychological safety | Lots of examples of hierarchy affecting psychological safety | N/A |
| Kaafarani et al, 2006 (43) | Moderate - found lower psychological safety within theatre & recovery than in other areas | Mixed themes - "asking for help is a sign of incompetence", however "in my unit, patient safety problems and errors are communicated to the right people so the problem can be corrected" | Perception of undesirable consequences for patient safety |
| Kessel et al, 2012 (33) | Examined relationship between psychological safety, knowledge sharing and creative performance | Found that higher psychological safety led to improved knowledge sharing and creative performance | Improvement of team creativity |
| Kobayashi et al, 2005 (32) | Examined for cultural differences between Japanese and US residents - none found. | Psychological safety affected by climate, but that organisational and professional culture can override national culture | Perception of undesirable consequences for patient safety |
| Kolbe et al, 2012 (78) | Variable - dependent upon role. | Nurses spoke up more than residents in the simulated scenarios. Were more likely to speak up as the scenario progressed if had spoken up at the start. | Positive relationship between speaking up behaviour and technical team performance |
| Law et al, 2014 (79) | Variable - improved when empowered by supportive seniors and peers to speak up | Benefits of mentors speaking up and leading by example, but that this can also be negative as it suggests you need to be senior to speak up. Speaking up avoided to avoid interpersonal conflict. |  |
| Livorsi et al, 2014 (34) | Low | Barriers included: fear of repercussion, unclear expectations, knowledge deficits and a culture of negativity. | Perception of undesirable consequences for patient safety |
| Lockett et al, 2015 (45) | High levels of psychological safety, but lots of barriers identified | Recognition that speaking up is an ethical responsibility "to uphold acceptable standards of behaviour and patient care" Barriers Included: Blame culture, current workload, fear of conflict | N/A |
| Lyndon et al, 2012 (64) | Low - even if harm rating high | Harm rating was a predictor of speaking up. Likelihood of speaking up correlated with scores for bravery and assertiveness. Shared mental model crucial for promoting psychological safety. Intimidation by seniors barrier to psychological safety. | Perception of undesirable consequences for patient safety |
| Malloy et al, 2009 (80) | Low, but some variation according to culture. | Lack of empowerment and psychological safety related to hierarchy. | Moral Distress in Nurses as a consequence of acting out decisions they are not involved in |
| Martinez et al, 2014 (29) | Generally high | Higher willingness to speak up about patient safety concerns as opposed to unprofessional behaviour. Barriers included: fear of conflict, alienation from team members. Facilitators included "evidence that speaking up will lead to meaningful change" | N/A |
| Maxfield et al, 2013 (65) | Low | Paper demonstrated evidence of organisational silence. Barriers included: conflict, fear of retaliation, poor previous experiences | Perception of undesirable consequences for patient safety and difficulty maintaining quality and safety |
| McLinton et al, 2017 (81) | Moderate - but demonstrated room for improvement | Highlighted a need for a positive culture and the crucial role of management in this. Low psychological safety when managers fail to values staff. | N/A |
| Nembhard et al, 2005 (68) | Moderate | Longevity and experience associated with a higher level of psychological safety. Low leader inclusiveness a barrier to psychological safety | N/A |
| Ortega et al, 2011 (44) | Assessed association between psychological safety and team learning | Team beliefs about psychological safety were positively associated with team learning. Poor team learning and negative environment associated with low psychological safety | N/A |
| Pian-Smith, 2009 (53) | Improvement post intervention: simulated encounters and teaching of "two-challenge rule" | Instructional intervention led to an improvement in speaking up - particularly in senior doctors | N/A |
| Piers et al, 2017 (61) | Both high and low levels of psychological safety reported. | Organisational culture crucial for higher psychological safety | N/A |
| Putnam et al, 2015 (72) | Low | Only 30% agreed with the statement "I feel free to question the decisions or actions of those with more authority". Nurses scored more highly for speaking up than residents; and junior residents demonstrated increased willingness compared to senior residents. | N/A |
| Raemer et al, 2016 (86) | Assessed impact of an intervention. No significant different pre or post intervention | "Speaking up behaviours are deeply rooted and difficult to change, at least with education alone. Barriers included: uncertainty, familiarity with team, expected repercussions. | N/A |
| Rathert et al, 2009 (46) | Assessed factors associated with psychological safety | Those with greater continuous quality improvement reported greater psychological safety. | Perception of undesirable consequences for patient safety |
| Richard et al, 2017 (30) | Both high and low levels of psychological safety reported. | Different motivations for silence and speaking up - study describes these as separate, multidimensional concepts. | High psychological safety has a positive impact on teamwork, personal engagement and seeking feedback. |
| Roberts et al, 2014 (31) | Explored factors promoting psychological safety | Previous positive experiences increased likelihood of speaking up. Barriers: concern regarding response from seniors. | N/A |
| Roussin et al, 2018 (35) | High | Factors promoting psychological safety: Female sex, increased age. Those with greater levels of occupational self-efficacy. | N/A |
| Rutherford et al, 2012 (82) | Moderate - majority would speak up | Theme of using an intermediary to escalate concerns emerged. | N/A |
| Sayre et al, 2012 (54) | Statistically significant improvement in speaking up for those who had undergone the intervention | By discussing patient scenarios, individuals reported an increased number of behaviour options. "An educational intervention can increase nurse’s perception of their own abilities to speak up and collaborate" | N/A |
| Schwappach and Gering, 2014 (36) | Moderate | Facilitators: Obligation to speak up for patient safety, and also protect the individual who made the error. Barriers: Not wanting to "humiliate anyone" | Perception of undesirable consequences for patient safety |
| Schwappach and Gering, 2014 (37) | Both high and low levels of psychological safety reported. | Potential harm was a strong predictor of the likelihood of speaking up. Barriers identified included: Male sex, younger age, hierarchy | N/A |
| Schwappach and Richard, 2018 (83) | Low - both in terms of withholding voice and speaking up | Encouraging environment was significantly positively associated with speaking up and a lower likelihood of withholding voice. Strong influence of hierarchy on speaking up behaviour | N/A |
| Schwappach and Sendlhofer (47) | Compared speaking up in Academic and Non-academic Centres - higher in academic sites | Most important factor promoting psychological safety: Perceived risk of harm to patient. Same barriers to psychological safety in academic and non-academic sites, but less discomfort felt regarding speaking up in academic sites. | N/A |
| Schwappach et al, 2018 (66) | Moderate to high levels of psychological safety | Higher risk of harm and higher frequencies of past speaking up behaviours were positively associated with likelihood to speak up | N/A |
| Schwappach and Sendlhofer, 2018 (48) | Both high and low levels of psychological safety reported. | Psychological safety lower for healthcare workers with managerial functions | N/A |
| Sexton et al, 2006 (62) | Both high and low levels of psychological safety reported. | Nurses were less positive about speaking up, feeling supported by others, physician-nurse collaboration, conflict resolution and heeding nurse input | N/A |
| Sundqvist et al, 2013 (84) | Moderate | Facilitator: Obligation to act as the patients advocate. Speaking up seen as difficult but an “easy burden to bear” as they felt they had done good for the patient | Increased self-esteem associated with psychological safety and good teamwork |
| Sur et al, 2015 (69) | High - majority recalled experiences of speaking up | Concept of measurable harm prompting people to speak up. Factors affecting willingness to speak up: systemic factors, supervisor factors, trainee factors and clinical factors. Decreased willingness to speak up was associated with the perceptions of supervisors as less approachable, less familiar, more senior and more experienced | N/A |
| Szymczak et al, 2015 (67) | Both high and low levels of psychological safety reported. | Consideration of harm to patient from breach in practice, but also potential harm to patient that may be caused by conflict within the team. Importance of team members being in agreement. Significance of behaviour being influenced by previous experiences. | N/A |
| Tamuz et al, 2011 (85) | Moderate - demonstrated room for improvement | Barriers included: embarrassment, concern about "looking stupid or weak". Nurses tended to use more direct language than physicians. Use of deferential language in order to enable raising concerns to seniors. | N/A |
| Tangirala et al, 2008 (38) | Both high and low levels of psychological safety reported. | The relationship between personal control and voice was "u-shaped" – voice was higher at high and low levels of personal control. Individuals who identify with the organisation more likely to speak up | N/A |
| Tarrant et al, 2017 (39) | Both high and low levels of psychological safety reported. | Hierarchy a barrier to speaking up. Some physicians reported that direct challenge in the other direction was potentially uncomfortable – fear that the challenge might be interpreted as bullying. “I certainly would find it much easier to challenge one of my medical colleagues than I would a nurse” | N/A |
| Todorova et al, 2012 (59) | Low | Reluctance to speak up as a consequence of prior negative experiences | N/A |
| Urisman et al, 2018 (55) | Large improvement following intervention (Change in ward round format) | Improvement in communication due to improved feelings of inclusion by all team members during rounds | N/A |
| Van Bogaert et al, 2015 (87) | Positive impact of structural empowerment | Improved communication and decision making and feelings of empowerment. | N/A |
| Weiss et al, 2014 (76) | High proportion of participants remained silent even if it would cause a highly critical event | Need to balance between work relationships and patient care. Nurses more likely to speak up to a member of their own team, unlike physicians who would speak up to surgeon | N/A |
| Weller et al, 2011 (73) | Both high and low levels of psychological safety reported. | Need for acknowledgement of individuals contributions to promote future psychological safety. Also identifies problems associated with being "too approachable". | N/A |
| Whitehair et al, 2018 (63) | Moderate | Teamwork requires an individual’s confidence in their own abilities. Highlights importance of continual learning and training in successful teamwork. | N/A |
