# Supplementary material for: The presence and potential impact of psychological safety in the healthcare setting: an evidence synthesis
Source: BMC Health Serv Res. 2021 Aug 5;21:773. doi: 10.1186/s12913-021-06740-6 (PMC8344175; doi:10.1186/s12913-021-06740-6)
Supplement: Supplementary file 3 — Additional file 3: Supporting Quotes: Experiences of Psychological Safety. [file 12913_2021_6740_MOESM3_ESM.docx]

**ADDITIONAL FILE 3:**

**Supporting Quotes: Experiences of Psychological Safety**

| **EXAMPLES OF LOW PSYCHOLOGICAL SAFETY** | | **EXAMPLES OF HIGH PSYCHOLOGICAL SAFETY** | |
| --- | --- | --- | --- |
| “you speak and then you keep getting this resistance or this stifling or this intimidation factor and then people start shutting down” | Garon et al (74) | “I am rewarded for taking quick action to identify a serious mistake” | Kaafarani et al (43) |
| “Do we feel free to talk? Absolutely. Does it go anywhere? No” | Garon et al (74) | *“*in my unit, patient safety problems and errors are communicated to the right people so the problem can be corrected” | Kaafarani et al (43) |
| “asking for help is a sign of incompetence” | Kaarfarani et al (43) | “unit culture was characterised as having a sense of camaraderie or teamwork with trust and respect” | Lockett et al (45) |
| “nothing ever changes so I have stopped trying” | Lockett et al (45) | “somehow find the strength to dare to speak out and dare to question” | Sundqvist et al (84) |
| *“a lot of people are still in awe of physicians and will not question physicians”* | Lyndon et al (64) | *“we’d done a timeout, we knew each other’s names, we were all focused on the same thing”* | Sur et al (69) |
| “you have many battles to fight with multiple disciplines and that gets exhausting so you learn to pick your battles” | Szymczak et al (67) | “he was extremely grateful and thanked me profusely in front of everyone…it made me feel so empowered to speak up again” | Sur et al (69) |
| *“there is nowhere to turn. They [management] just laugh at you or look through you”* | Torodova et al (59) | “I would certainly find it much easier to challenge one of my medical colleagues than I would a nurse” | Tarrant et al (39) |
|  |  | *“everyone’s view is listened to, even if it’s in the minority”* | Hirak et al (57) |
|  |  | “There are real attempts to share information throughout the unit” | Hirak et al (57) |
