# Supplementary material for: The presence and potential impact of psychological safety in the healthcare setting: an evidence synthesis
Source: BMC Health Serv Res. 2021 Aug 5;21:773. doi: 10.1186/s12913-021-06740-6 (PMC8344175; doi:10.1186/s12913-021-06740-6)
Supplement: Supplementary file 4 — Additional file 4: Possible consequences of high or low psychological safety. [file 12913_2021_6740_MOESM4_ESM.docx]

**ADDITIONAL FILE 4: Possible consequences of high or low psychological safety**

| **Paper** | **Research Methodology** | **Aims** | **Analysis** | **Potential Consequence Identified** | **Nature of relationship** | **Causal or Correlational** |
| --- | --- | --- | --- | --- | --- | --- |
| Kessel et al (1) | Quantitative - Survey | "Examine psychological safety as a mediator of the relationship between knowledge sharing and creative performance" | Latent class regression analysis | Positive relationship between psychological safety and creative performance (p <0.05) | Statistically significant | Causal (authors state that lack of experimental design means this cannot be ensured) |
|  |  |  |  | Positive relationship between psychological safety and knowledge sharing (p <0.05) | Statistically significant | Causal (authors state that lack of experimental design means this cannot be ensured) |
| Kolbe et al (2) | Quantitative - Simulator, Prospective observational study | "To test the relationship between speaking up and technical team performance in anaesthesia" | Linear regression | Positive relationship between speaking up behaviour and technical team performance in nurses (p=<0.017) | Statistically significant | Correlational design |
| Richard et al (3) | Quantitative - Survey | "To develop a short questionnaire allowing healthcare organisations to assess different aspects of speaking up among healthcare staff" | Principle component analysis | Healthcare workers without managerial responsibilities had lower psychological safety for speaking up than those with managerial roles. | Statistically significant | Correlational |
|  |  |  | Content validity | Psychological safety for speaking up was negatively correlated with withholding voice and speaking up (-0.53, -0.23) | Statistically significant | Correlational |
| Sundqvist et al (4) | Qualitative - Interview | "The aim of the study was to describe advocacy in anaesthesia care during the perioperative phase from the perspective of the registered nurse anaesthetist" | Qualitative Content Analysis | Increased self-esteem associated with speaking up and good teamwork (markers of psychological safety) | Qualitative Theme | Correlational |
| Aveling et al (5) | Qualitative - Interview | "Explore how healthcare workers in two East African hospitals identify and explain the major obstacles to ensuring the safety of patients in their care." | Qualitative Thematic Analysis | Highlights many factors associated with low psychological safety and links these to poor patient outcomes | Qualitative Theme | Causal - perceived by authors |
| Belyansky et al (6) | Quantitative - Survey | "Examine the factors that influence surgical trainees in expressing their opinion in the operating room and the consequences this might have on patient safety" | Chi Squared, Fisher exact tests and Wilcoxon rank sum test | Communication between resident and attending can prevent adverse patient events, influenced negatively by hierarchy | Reported by study participants | Causal - perceived by authors |
| Garon et al (7) | Qualitative - Focus Group | "To explore nurses’ perceptions of their own ability to speak up and be heard in the workforce" | Thematic Content Analysis | Infer that low psychological safety can be harmful for patients | Qualitative Theme | Causal - perceived by authors |
| Garon et al (8) | Qualitative - Interview | "To relate nurses’ stories of their experiences of acts of resistance" | Narrative Analysis | Highlights many factors associated with low psychological safety and links these to poor patient outcomes, retaliation and financial impact | Qualitative Theme | Causal - perceived by authors |
|  |  |  |  | Positive relationship between psychological safety and feelings of empowerment and organisational change | Qualitative Theme | Causal - perceived by authors |
| Kaafarani et al (9) | Quantitative - Survey | "To evaluate patient safety culture in the OR and PACU" | Measured percent problematic response | Infer that low psychological safety and poor communication can be detrimental to patient safety | Reported by study participants | Causal - perceived by authors |
| Kobayashi et al (10) | Quantitative - Survey | "To identify perceived barriers to residents’ questioning or challenging their seniors, to determine how these barriers affect decisions and to assess how these barriers differ across cultures" | Descriptive Statistics | Inference that participants would not speak up to prevent error | Reported by study participants | Causal - perceived by authors |
| Livorsi et al (11) | Qualitative - Interview | "To explore three safety domains: reporting errors, approachability of authority figures and handovers." | Thematic Analysis | Highlights many factors associated with low psychological safety and links these to poor patient outcomes | Qualitative Theme | Causal - perceived by authors |
| Lyndon et al (12) | Quantitative - Survey | "Assessment of likely harm and relationship to speaking up in response to perceived harm" | Descriptive statistics and bivariate analyses | Risk of harm to patients associated with low psychological safety | Inferred by authors | Causal - perceived by authors |
| Maxfield et al (13) | Quantitative - Survey | "To assess the occurrence of 4 safety concerns among labor and delivery teams: dangerous shortcuts, missing competencies, disrespect and performance problems." | Descriptive Statistics | Low psychological safety undermined patient safety, harmed patients and led to staff leaving | Reported by study participants | Causal - perceived by authors |
| Rathert et al (14) | Quantitative - Survey | "To empirically explore a theoretical model linking the work environment in the healthcare setting and how it might relate to work engagement, organisational commitment and patient safety" | Latent regression analysis | Continuous quality improvement significantly and positively related to psychological safety  Patient centred care significantly and negatively related to psychological safety | Statistically Significant | Correlational |
|  |  |  |  |  | Statistically Significant | Correlational |
| Schwappach et al (15) | Qualitative - Interview | "To investigate the motivations and barriers to speaking up towards co-workers and supervisors" | Thematic Content Analysis | Low psychological safety and reluctance to speak up linked to a negative impact on patient safety | Inferred by authors | Causal - perceived by authors |
| Hirak et al (16) | Quantitative - Survey | "Analyse how the behaviour of the leader influences psychological safety and learning from failure" | Descriptive and correlational statistics | Psychological safety climate positively associated with unit learning from failure (p<0.01) | Statistically significant | Correlational |
|  |  |  |  | Psychological safety climate positively associated with unit performance (p<0.05) | Statistically significant | Correlational |
| Malloy et al (17) | Qualitative - Focus Groups | "Identify ethical dilemmas in caring for elderly people with dementia" | Thematic Content Analysis | Low psychological safety risked moral distress for staff | Qualitative Theme | Causal - perceived by authors |
| Alilu et al (18) | Qualitative - Interview | "To explore professional challenges in clinical settings and reasons for wanting to leave the profession" | Thematic and Content analysis | Factors related to low psychological safety associated with increased intention to leave profession | Qualitative Theme | Causal - perceived by authors |

1. Kessel M, Kratzer J, Schultz C. Psychological safety, knowledge sharing, and creative performance in healthcare teams. Creativity and innovation management. 2012;21(2):147-57.

2. Kolbe M, Burtscher MJ, Wacker J, Grande B, Nohynkova R, Manser T, et al. Speaking up is related to better team performance in simulated anesthesia inductions: an observational study. Anesthesia & Analgesia. 2012;115(5):1099-108.

3. Richard A, Pfeiffer Y, Schwappach D. Development and Psychometric Evaluation of the Speaking Up About Patient Safety Questionnaire. Journal of patient safety. 2017.

4. Sundqvist AS, Carlsson AA. Holding the patient's life in my hands: S wedish registered nurse anaesthetists' perspective of advocacy. Scandinavian journal of caring sciences. 2014;28(2):281-8.

5. Aveling E-L, Kayonga Y, Nega A, Dixon-Woods M. Why is patient safety so hard in low-income countries? A qualitative study of healthcare workers’ views in two African hospitals. Globalization and health. 2015;11(1):6.

6. Belyansky I, Martin TR, Prabhu AS, Tsirline VB, Howley LD, Phillips R, et al. Poor resident-attending intraoperative communication may compromise patient safety. Journal of Surgical Research. 2011;171(2):386-94.

7. Garon M. Speaking up, being heard: registered nurses' perceptions of workplace communication. Journal of Nursing Management. 2012;20(3):361-71.

8. Garon M. The positive face of resistance: nurses relate their stories. JONA: The Journal of Nursing Administration. 2006;36(5):249-58.

9. Kaafarani HM, Itani KM, Rosen AK, Zhao S, Hartmann CW, Gaba DM. How does patient safety culture in the operating room and post-anesthesia care unit compare to the rest of the hospital? The American Journal of Surgery. 2009;198(1):70-5.

10. Kobayashi H, Pian-Smith M, Sato M, Sawa R, Takeshita T, Raemer D. A cross-cultural survey of residents' perceived barriers in questioning/challenging authority. Qual Saf Health Care. 2006;15(4):277-83.

11. Livorsi D, Knobloch M, Blue L, Swafford K, Maze L, Riggins K, et al. A rapid assessment of barriers and facilitators to safety culture in an intensive care unit. International nursing review. 2016;63(3):372-6.

12. Lyndon A, Sexton JB, Simpson KR, Rosenstein A, Lee KA, Wachter RM. Predictors of likelihood of speaking up about safety concerns in labour and delivery. BMJ Publishing Group Ltd; 2012.

13. Maxfield DG, Lyndon A, Kennedy HP, O'Keeffe DF, Zlatnik MG. Confronting safety gaps across labor and delivery teams. American journal of obstetrics and gynecology. 2013;209(5):402-8. e3.

14. Rathert C, Ishqaidef G, May DR. Improving work environments in health care: Test of a theoretical framework. Health care management review. 2009;34(4):334-43.

15. Schwappach DL, Gehring K. Silence that can be dangerous: a vignette study to assess healthcare professionals’ likelihood of speaking up about safety concerns. PLoS One. 2014;9(8).

16. Hirak R, Peng AC, Carmeli A, Schaubroeck JM. Linking leader inclusiveness to work unit performance: The importance of psychological safety and learning from failures. The Leadership Quarterly. 2012;23(1):107-17.

17. Malloy DC, Hadjistavropoulos T, McCarthy EF, Evans RJ, Zakus DH, Park I, et al. Culture and organizational climate: nurses’ insights into their relationship with physicians. Nursing Ethics. 2009;16(6):719-33.

18. Alilu L, Zamanzadeh V, Fooladi MM, Valizadeh L, Habibzadeh H. Towards an understanding of clinical nurses challenges that leads intention to leave. Acta Paulista De Enfermagem. 2016;29(5):534-41.
