# Supplementary material for: The presence and potential impact of psychological safety in the healthcare setting: an evidence synthesis
Source: BMC Health Serv Res. 2021 Aug 5;21:773. doi: 10.1186/s12913-021-06740-6 (PMC8344175; doi:10.1186/s12913-021-06740-6)
Supplement: Supplementary file 5 — Additional file 5: Facilitators and Barriers to Psychological Safety. [file 12913_2021_6740_MOESM5_ESM.docx]

**ADDITIONAL FILE 5: Facilitators and Barriers to Psychological Safety**

| **THEME** | FACILLITATORS | Examples of Supporting Data | BARRIERS | Examples of Supporting Data |
| --- | --- | --- | --- | --- |
| **CULTURE** | Creation of a positive culture by management  Leader inclusiveness  Positive environment  Encouragement of questions from senior staff  Enablement of nursing staff to be an active member of the team  Use of an intermediary to escalate concerns | *“everyone’s view is listened to, even if it’s in the minority”*    *“There are real attempts to share information throughout the unit” (1)*  *“a facilitating organisational culture being open, where raising concerns was perceived as a professional duty and responsibility” attree et al*  *“I trust my manager, I have absolute confidence in her. I am confident that my manager would support me totally” attree et al*  *“I think you set a culture for nurses to speak up…it seems like the timing and respectfulness and the openness is more available” baik et al*  *“usually they’ll sweep back to you and say ‘do you have any thoughts’ ‘did you have anything you wanted to add’?” baik et al* | Hierarchy and fear of challenging authority  Fear of repercussion: permanent record, “being shot down”  Lack of leader inclusiveness  Blame culture  Staff members feeling under-valued  Uncertainty of recipient’s response | *“you speak and then you keep getting this resistance or this stifling or this intimidation factor and then people start shutting down” (2)*  *“a nurse who demonstrates being more knowledgeable than a doctor (by speaking up) is seen as stepping outside of her role. If this were to happen, a surgeon would reinstate his hierarchy when the opportunity arises” (3)*  *“there is nowhere to turn. They [management] just laugh at you or look through you” (4)*  *“I think they look down on you “Abdi et al*  *“instead of thanking me, the supervisors reprimanded me, which I didn’t expect” Abdi et al*  *“Management only assigned blame, instead of finding a solution” alilu et al*  *“we’re the ones that get the blame for everything” attree et al*  *“they don’t even listen to you” Aveling et al*  *“many of us are very sad for the fact that physicians have such high status in the hospital, especially male doctors, and we [nurse] are treated as worthless” alilu et al* |
| **SAFETY CULTURE** | Use of policies and hospital documents  Enhanced safety curriculum  Enablement of nursing staff to be an active member of the team  Evidence of continuous quality improvement | *“nurses who perceive that their direct supervisor shows commitment and role modelling behaviour, creates awareness and encourages them to participate perceive the environment to be psychologically safe for taking interpersonal risks” alingh et al*  *“structured rounding format and communication tools…enabled nurses to be an active member of the team and provided a safe environment for them” baik et al* | Negative culture of conformity to instructions of senior nurses / doctors.  Lack of feedback from incident reporting | *“unit characterised as mistrustful, uncivil or negative”. (5)*  *“whenever an error happens, we could say that the person has been either incompetent or reckless” abdi et al*  *“they gave me a medication error reporting form which I filled and submitted, but I never received feedback and there was no follow up either” abdi et al.* |
| **WORKLOAD** | Manageable | *“I think [I feel] better job satisfaction because of my workflow…it’s a more effective use of my time” baik et al* | High | *“ you have many battles to fight with multiple disciplines and that gets exhausting so you learn to pick your battles” (6)*  *‘there is high levels of stress associated with accomplishing the tasks on time” alilu et al* |
| **INFRASTRUCTURE** | Policies & hospital documents  Good reporting systems  Private location for raising concerns | *“in my unit, patient safety problems and errors are communicated to the right people so the problem can be corrected” (7)* | Public location for raising concerns  Covering several locations | *“the public space is considered unsafe when the dynamic flow of people in the situation is unsupportive and unwilling to listen” law et al*  *“less likely to speak up towards the senior physician…in a public forum when several other HCPs are present” schappach & gehring 2014*  *“as the doctors and nurses in any team looking after a particular patient were all members of multiple and different care teams, this made it difficult for them to meet together at the same time to share information” weller et al*  *“because the nurses were with patients constantly, they felt they could pick up subtle changes but not always adequately convey their concerns to medical staff” weller et al* |
| **CONFIDENCE** | Positive Previous Experience of speaking up  Own clinical experience  Action resulting from previous episodes of speaking up  More senior professional status  Older Staff Members | *“I am rewarded for taking quick action to identify a serious mistake” (7)*  *“nurses willingness to speak is mainly driven by the confidence that raising patient safety concerns will not have negative personal consequences” alingh et al*  *“raising concerns would depend on individuals, the level of the nurse, how strongly they felt it was unacceptable and how assertive and confident they were in themselves to be able to raise it” attree et al* | Negative previous experience  Fear of being disrespectful  Fear of losing professional respect  Fear of challenging authority  Lack of feedback from previous experiences of speaking up  Fear of managing potential conflict  Uncertainty regarding issue to be raised  Fear of repercussion | *“fear that negative consequences would result” attree et al*  *“indecision and hesitancy were attributed to fear of repercussions, retribution, labelling and blame for raising concerns” attree et al*  *“low likelihood of positive action resulting from raising concerns” attree et al*  *“they feel that nothing is being done, sometimes you get a response or feedback and sometimes – often you don’t” attree*  *“feeling self-conscious about asking questions about their patients” baik et al* |
| **OCCUPATIONAL SELF EFFICACY** | High | *“participants with greater occupational self-efficacy were significantly more likely to speak up when unclear about something explained” roussin et al* | Low |  |
| **TEAMWORK** | Shared Mental Model  Supportive seniors  Common goals for patient care  Familiarity with team members | *“was lucky that I felt comfortable approaching the anaesthetist because I knew who he was” (8)*  *“unit culture was characterised as having a sense of camaraderie or teamwork with trust and respect” (5)*  *“knowing and understanding long-term plans for patient care would be helpful” baik et al* | Retaliation from colleagues  Alienation from team  Unclear expectations  Lack of familiarity | *“I think there are doctors who do not know the real meaning of teamwork…they don’t realise that our viewpoint, as members of the medical team, should also be considered” abdi et al*  *“in some wards there is envy, jealousy, bickering and backstabbing among the healthcare providers, creating a negative atmosphere with conflicts” alilu et al*  *“trust takes time to develop…you don’t really know the people there and you don’t know if you can trust them” weller et al* |
| **ABILITY** | Level of Clinical Experience  Communication Skills | *“strongly determined by seniority” schwappach & gehring 2014* | Knowledge deficit  Communication skills  Unconfident in clinical skills | *“asking for help is a sign of incompetence” (7)* |
| **MOTIVATION** | Patient safety Concerns  Risk of serious harm | *“Fighting for our patients”.(2)*  *“there are times when a patient’s life is at risk and I could not remain silent” abdi et al*  *“they have a professional duty to do something” attree et al* | Unprofessional behaviour  Professional concerns | *“if I think the risk to the patient is relatively low…that’s probably something I wouldn’t be as aggressive about speaking up on”(9)*  *“The problem had to be big enough to motivate them to speak up” (5)*  *“some prefer not to do so for legal reasons” abdi et al* |

1. Hirak R, Peng AC, Carmeli A, Schaubroeck JM. Linking leader inclusiveness to work unit performance: The importance of psychological safety and learning from failures. The Leadership Quarterly. 2012;23(1):107-17.

2. Garon M. Speaking up, being heard: registered nurses' perceptions of workplace communication. Journal of Nursing Management. 2012;20(3):361-71.

3. Jayasuriya-Illesinghe V, Guruge S, Gamage B, Espin S. Interprofessional work in operating rooms: a qualitative study from Sri Lanka. BMC surgery. 2016;16(1):61.

4. Todorova IL, Alexandrova‐Karamanova A, Panayotova Y, Dimitrova E. Organizational hierarchies in B ulgarian hospitals and perceptions of justice. British journal of health psychology. 2014;19(1):204-18.

5. Lockett JJ, Barkley L, Stichler J, Palomo J, Kik B, Walker C, et al. Defining peer-to-peer accountability from the nurse’s perspective. JONA: The Journal of Nursing Administration. 2015;45(11):557-62.

6. Szymczak JE. Infections and interaction rituals in the organisation: clinician accounts of speaking up or remaining silent in the face of threats to patient safety. Sociology of health & illness. 2016;38(2):325-39.

7. Kaafarani HM, Itani KM, Rosen AK, Zhao S, Hartmann CW, Gaba DM. How does patient safety culture in the operating room and post-anesthesia care unit compare to the rest of the hospital? The American Journal of Surgery. 2009;198(1):70-5.

8. Rutherford J, Flin R, Mitchell L. Teamwork, communication, and anaesthetic assistance in Scotland. British journal of anaesthesia. 2012;109(1):21-6.

9. Sur MD, Schindler N, Singh P, Angelos P, Langerman A. Young surgeons on speaking up: when and how surgical trainees voice concerns about supervisors' clinical decisions. The American Journal of Surgery. 2016;211(2):437-44.
